# Hypertrophic Obstructive Cardiomyopathy During Pregnancy: A Report of Two Cases

**DOI:** 10.7759/cureus.102304

**Published:** 2026-01-26

**Authors:** Pedro A Román, Carlos M Penate, Gleiny Vázquez, Carlos Fonseca, Román Vasallo

**Affiliations:** 1 Heart Disease and Pregnancy, Hospital Materno Ramón González Coro, Havana, CUB; 2 Cardiology, Instituto de Cardiología y Cirugía Cardiovascular (ICCCV), Havana, CUB

**Keywords:** cardiomyopathy, hemodynamic, hypertrophic, obstructive, pregnancy

## Abstract

Hypertrophic cardiomyopathy (HCM) is the most common genetic cardiomyopathy caused by pathogenic variants in sarcomeric proteins and defined by left ventricular (LV) wall thickness ≥15 mm at end diastole in the absence of abnormal loading conditions. Hypertrophic obstructive cardiomyopathy (HOCM) is the obstructive phenotype of HCM characterized by dynamic left ventricular outflow tract* *(LVOT) obstruction, most often driven by septal hypertrophy and systolic anterior motion (SAM) of the mitral valve, frequently with mitral regurgitation. Pregnancy is generally well-tolerated in women with HCM; however, outcomes may be worse when significant left ventricular outflow tract obstruction (LVOTO) is present. Pregnancy-related increases in heart rate and reductions in systemic vascular resistance may intensify dynamic LVOTO, particularly when preload or afterload decreases. In severe symptomatic cases, pregnancy may be contraindicated (modified World Health Organization (mWHO) class IV, an extremely high-risk category in which pregnancy is generally discouraged) and therefore requires individualized counseling and specialized multidisciplinary care. This report describes two cases (a two-patient case series) of pregnant women with HOCM who required close hemodynamic monitoring and optimized peripartum management.

## Introduction

Hypertrophic cardiomyopathy (HCM) is the most prevalent genetic cardiomyopathy resulting from pathogenic variants in sarcomeric proteins, defined by a left ventricular (LV) wall thickness greater than 15 mm in the absence of any other cause of hypertrophy [1]. The prevalence of HCM in women planning pregnancy has increased in recent years due to the use of more diagnostic tools and family screening, with reported prevalence estimates ranging from 1 in 1,000 to 1 in 5,000 [2,3]. About 70% of patients with HCM develop left ventricular outflow tract obstruction (LVOTO), often resulting from systolic anterior motion (SAM) of the mitral valve and mitral regurgitation (MR) [4]. During pregnancy, several hemodynamic alterations involving cardiovascular changes occur, characterized by a cardiac output rise of approximately 50%, triggered by elevated heart rate (HR) and stroke volume, while systemic vascular resistance (SVR) decreases, resulting in a high-volume, low-resistance condition, accompanied by a reduction in blood pressure [5]. In contrast to non-obstructive HCM, hypertrophic obstructive cardiomyopathy (HOCM) is associated with greater maternal risk because dynamic LVOTO may intensify with tachycardia and with decreases in preload or afterload [6]. Additionally, during labor, whether it's a vaginal birth or a cesarean section, LVOTO can get worse due to increased heart rates or lower systemic vascular resistance, particularly when using general or epidural anesthesia [7]. Previous studies suggest that most pregnant women with non-obstructive HCM manage pregnancy successfully; however, those with HOCM face a higher risk of hemodynamic decompensation, including heart failure, thromboembolic events, and arrhythmias, especially when there is significant LVOTO [8]. Furthermore, many standard therapies for HOCM are contraindicated or not recommended during gestation [9]. We present a case series of pregnant women with HOCM.

## Case presentation

Case 1

**Figure 1:**
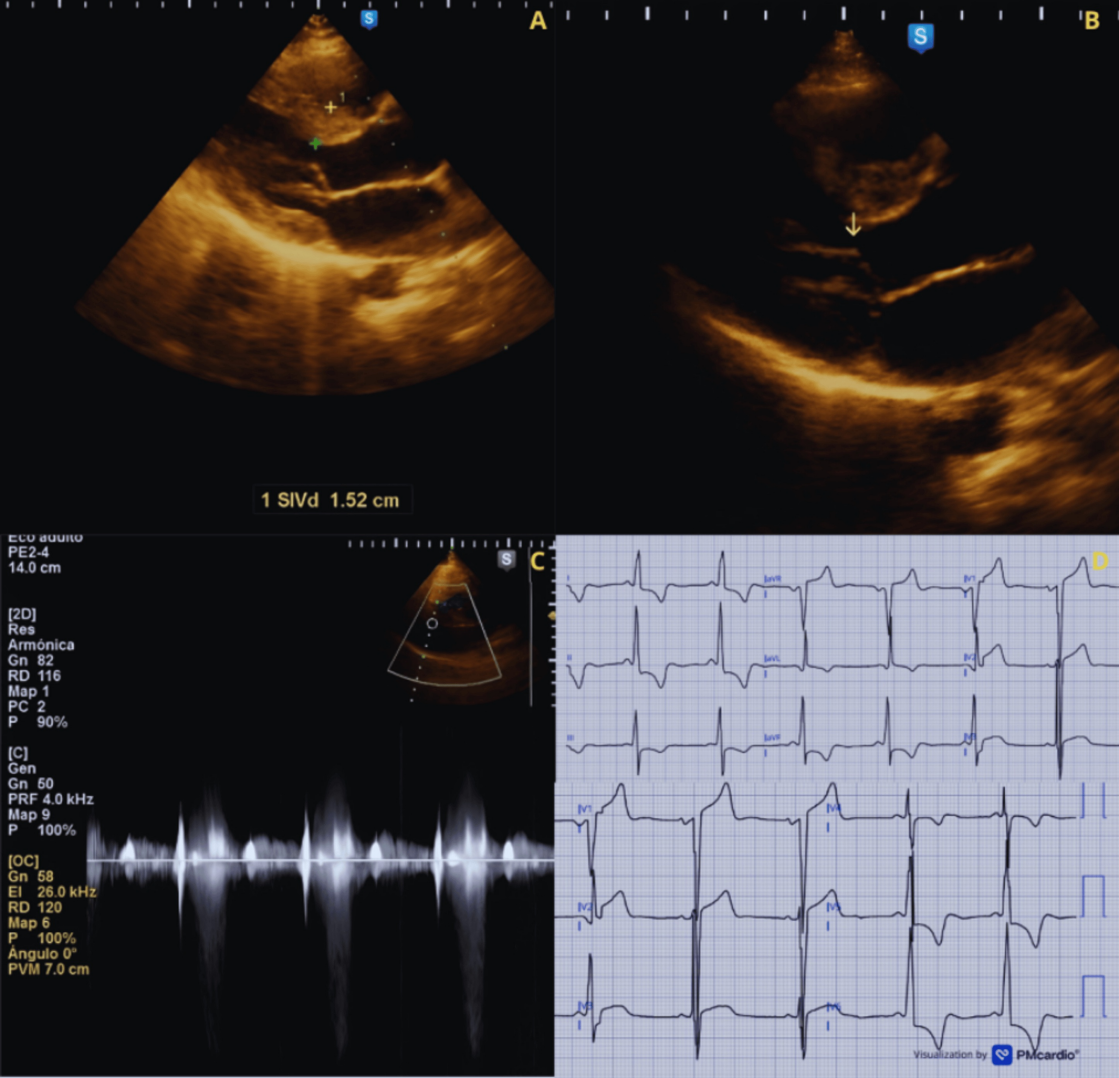
Echocardiographic and electrocardiographic features of Case 1 (A) Parasternal long-axis view showing asymmetric LVH predominantly involving the interventricular septum (1.52 cm = 15.2 mm). (B) Parasternal long-axis view showing systolic anterior motion (SAM) of the mitral valve (arrow) with dynamic left ventricular outflow tract (LVOT) narrowing. (C) Continuous-wave Doppler demonstrating a late-peaking systolic jet consistent with severe dynamic left ventricular outflow tract obstruction (LVOTO) (Dagger Sign). (D) ECG showing sinus rhythm with voltage criteria for left ventricular hypertrophy and repolarization abnormalities.

A 21-year-old woman (G1P000) with a history of HCM presented at 18 weeks of gestation. The patient reported dyspnea on moderate exertion, classified as New York Heart Association (NYHA) functional class II. On admission, vital signs were BP 115/70 mmHg, HR 85 bpm, RR 14/min, and temperature 36 °C. Physical examination revealed a grade III/VI mid-systolic murmur at the mid-precordium, increasing in intensity after Valsalva. The electrocardiogram showed a sinus rhythm and signs of LVH. The echocardiogram showed an asymmetric left ventricular hypertrophy predominantly involving the interventricular septum (15.2 mm), systolic anterior motion causing severe LVOTO, and moderate MR (Figure 1). Treatment with propranolol 60 mg once daily was initiated, with evident improvement in the gradients and mitigation of the symptoms. A cesarean section was performed at 39 weeks for cardiovascular indications, resulting in a newborn with Apgar scores of 9/9 and a weight of 3400 g. The patient remained hemodynamically stable during the postpartum period. 

Case 2

**Figure 2:**
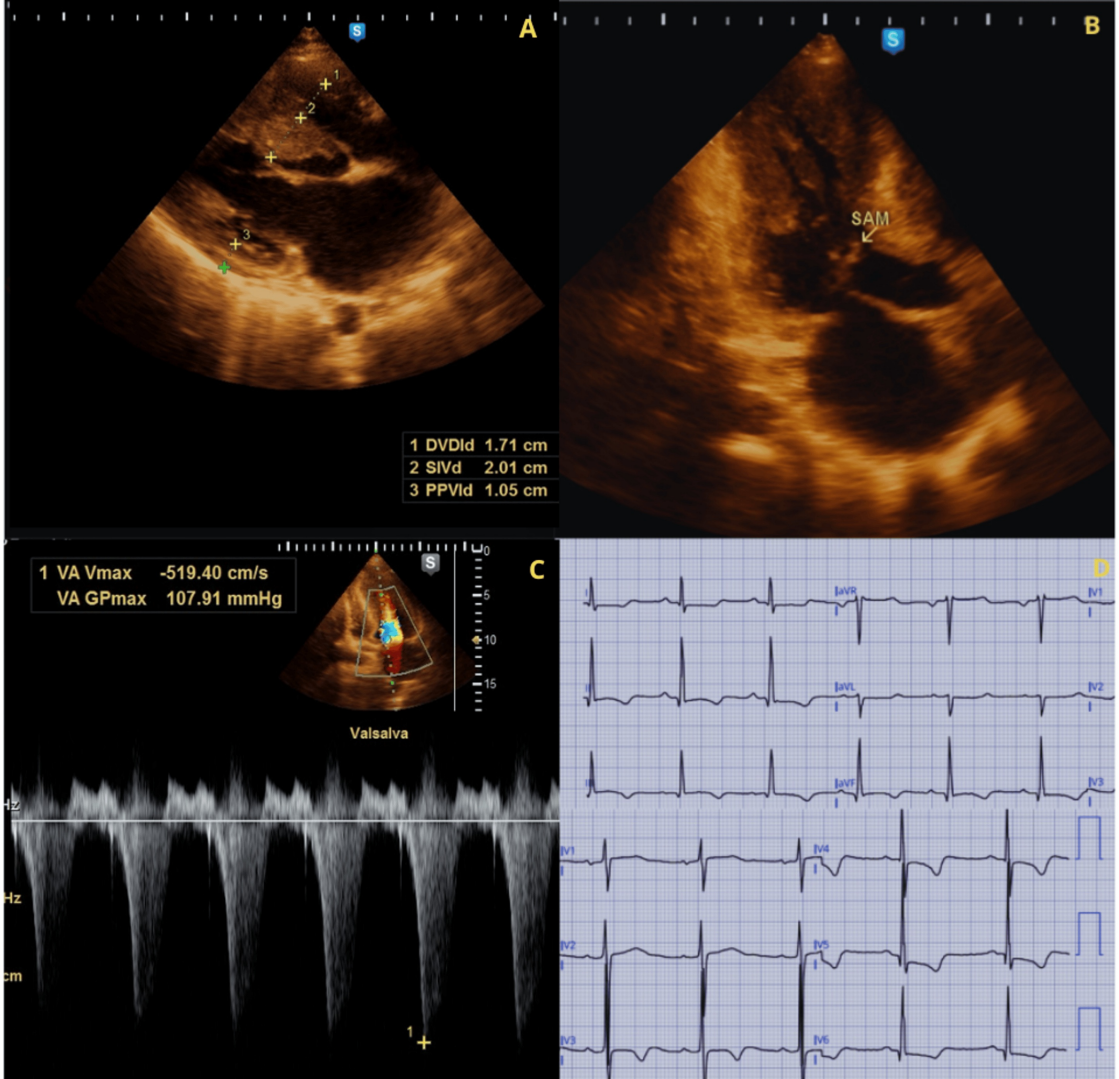
Echocardiographic and electrocardiographic findings of Case 2 (A) Parasternal long-axis view showing septal hypertrophy predominantly involving the interventricular septum (2.01 cm = 20.1 mm). (B) Apical three-chamber view showing systolic anterior motion (SAM) of the mitral valve (arrow) with dynamic left ventricular outflow tract narrowing. (C) Continuous-wave Doppler demonstrating a late-peaking systolic jet consistent with severe dynamic left ventricular outflow tract obstruction (LVOTO)(Dagger Sign). (D) ECG showing sinus rhythm with voltage criteria for left ventricular hypertrophy and repolarization abnormalities.

A 25-year-old woman (G2P0010) with a history of HCM and asthma presented at 17 weeks of gestation. The patient reported dyspnea and fatigue on moderate exertion, classified as New York Heart Association (NYHA) functional class II. On admission, vital signs were BP 120/80 mmHg, HR 87 bpm, RR 16/min, and temperature 36 °C. Physical examination revealed a grade III/VI mid-systolic murmur that increased with the Valsalva maneuver. The electrocardiogram showed sinus rhythm and signs of left ventricular hypertrophy. The echocardiogram showed a septal hypertrophy predominantly involving the interventricular septum (20.1 mm), systolic anterior motion causing severe LVOTO, and moderate MR (Figure 2). Verapamil 120 mg once daily was started, with a mild reduction in gradients and mitigation of the symptoms. A cesarean section was performed at 40 weeks for cardiovascular indications, resulting in a newborn with Apgar scores of 9/9 and a weight of 3200 g. During epidural anesthesia, she developed hypotension (75/60 mmHg), which resolved with phenylephrine infusion (0.2 µg/kg/min). The patient remained hemodynamically stable during the postpartum period.

## Discussion

HOCM is especially poorly tolerated owing to the hemodynamic changes that occur during pregnancy and is related to high maternal and fetal morbidity [6]. Prior studies showed that women with HCM have more severe disease expression and a 28% incidence of maternal complications, such as heart failure (HF), thromboembolic events, and arrhythmias, particularly in those with significant LVOTO [10]. Despite this potential impact, the mortality rate among pregnant women remains low [8,9,11]. Preconception counseling and risk stratification are crucial in women with HCM, particularly when LVOTO is present. A baseline evaluation should include symptom assessment (New York Heart Association (NYHA) functional class), electrocardiography, and transthoracic echocardiography with quantification of resting and provocable LVOT gradients, as well as assessment of mitral regurgitation and ventricular systolic function. Current European Society of Cardiology (ESC) guidelines recommend that women with severe symptomatic HOCM be counseled that pregnancy is contraindicated, as this scenario is classified as modified World Health Organization (mWHO) class IV, the highest maternal cardiovascular risk category [9]. Data from The Registry Of Pregnancy And Cardiac Disease (ROPAC) registry indicated that women classified as mWHO class IV have elevated rates of cardiovascular events [2]. Medical therapies are limited for patients with symptomatic HOCM and pregnancy; beta-blockers (BB), excluding atenolol (Food and Drug Administration pregnancy category D), or non-dihydropyridine calcium channel blockers (ND-CCBs) constitute first-line treatment [1,12]. However, the treatment must be adjusted with maternal-fetal monitoring, given the risk of fetal growth restriction, neonatal hypoglycemia, and fetal or neonatal bradycardia [8,11]. Unfortunately, for those who remain symptomatic or do not tolerate BB or ND-CCBs, mavacamten, a cardiac myosin inhibitor, is contraindicated due to fetal toxicity demonstrated in animal studies [13]. Also, disopyramide is avoided because it may stimulate uterine contractions [14]. These challenges emphasize the important role of preconception management, medication evaluation, and risk assessment. Vaginal delivery is often the primary choice for women without obstruction; however, cesarean delivery may be preferred if severe LVOTO is present to avoid abrupt hemodynamic decompensation [2,3,9]. β-agonist tocolytics can increase heart rate and contractility and may worsen dynamic LVOTO; therefore, they should be used with extreme caution or avoided in women with obstructive HCM [9]. Furthermore, epidural or general anesthesia can cause hypotension, which may exacerbate obstruction. Alpha-adrenergic vasopressors, such as phenylephrine, are preferred in this setting, as they increase afterload without increasing HR or myocardial contractility [15]. Although both patients had HOCM, their hemodynamic responses differed. In Case 2, the use of epidural anesthesia during the cesarean section caused hypotension that required an alpha-agonist, whereas Case 1 remained stable throughout the pregnancy. These cases emphasize the importance of a multidisciplinary approach to the management of high-risk cardiac conditions throughout pregnancy. This report is limited by the small sample size (two cases) from a single center, which restricts generalizability and precludes inference regarding incidence or comparative outcomes; nevertheless, it highlights practical hemodynamic and anesthetic principles relevant to severe LVOTO during pregnancy.

## Conclusions

Pregnancy in women with HOCM represents a high-risk clinical scenario requiring a multidisciplinary approach with the objective of preventing worsening obstruction and symptoms. Optimal management requires maintaining preload, avoiding tachycardia, and preserving afterload, which are fundamental principles of care. Preconception evaluation, as well as coordinated management throughout pregnancy, delivery, and the postpartum period, should be undertaken by a multidisciplinary team with experience. These two cases demonstrate how appropriate, anticipatory management can mitigate adverse outcomes in women with HOCM.

## References

[1] Ommen SR, Ho CY, Asif IM (2024). 2024 AHA/ACC/AMSSM/HRS/PACES/SCMR guideline for the management of hypertrophic cardiomyopathy: a report of the American Heart Association/American College of Cardiology joint committee on clinical practice guidelines. Circulation.

[2] Goland S, van Hagen IM, Elbaz-Greener G (2017). Pregnancy in women with hypertrophic cardiomyopathy: data from the European Society of Cardiology initiated Registry of Pregnancy and Cardiac disease (ROPAC). Eur Heart J.

[3] Alameh A, Jabri A, Sukhon F, Alhuneafat L, Arce PS, Siraj A (2024). Hypertrophic cardiomyopathy in pregnancy: a retrospective study of patients characteristics and outcomes. J Card Fail.

[4] Braunwald E (2025). Hypertrophic cardiomyopathy. N Engl J Med.

[5] Sanghavi M, Rutherford JD (2014). Cardiovascular physiology of pregnancy. Circulation.

[6] Thaman R, Varnava A, Hamid MS (2003). Pregnancy related complications in women with hypertrophic cardiomyopathy. Heart.

[7] Meng ML, Arendt KW, Banayan JM (2023). Anesthetic care of the pregnant patient with cardiovascular disease: a scientific statement from the American Heart Association. Circulation.

[8] Abdeldayem J, Abdelfattah OM, Chaabo O (2025). Long-term impact of pregnancy on clinical outcomes in individuals with hypertrophic cardiomyopathy. JACC Adv.

[9] De Backer J, Haugaa KH, Hasselberg NE (2025). 2025 ESC Guidelines for the management of cardiovascular disease and pregnancy. Eur Heart J.

[10] Geske JB, Ong KC, Siontis KC (2017). Women with hypertrophic cardiomyopathy have worse survival. Eur Heart J.

[11] Autore C, Conte MR, Piccininno M (2002). Risk associated with pregnancy in hypertrophic cardiomyopathy. J Am Coll Cardiol.

[12] Lydakis C, Lip GY, Beevers M (1999). Atenolol and fetal growth in pregnancies complicated by hypertension. Am J Hypertens.

[13] Bristol-Myers Squibb Company (2024). Bristol-Myers Squibb Company. CAMZYOS (mavacamten) prescribing information. Princeton, NJ: Bristol-Myers Squibb.

[14] Tadmor OP, Keren A, Rosenak D (1990). The effect of disopyramide on uterine contractions during pregnancy. Am J Obstet Gynecol.

[15] Mithani M, Flatow G, Chyfetz MA (2021). Management of a critically ill patient with severe hypertrophic obstructive cardiomyopathy presenting for emergent craniotomy due to subdural hemorrhage. Cureus.

